# 17th International Conference on Human Retroviruses: HTLV and Related Viruses, Trois Ilets, Martinique,(FWI). 18-21 June 2015

**DOI:** 10.1186/1742-4690-12-S1-I1

**Published:** 2015-08-28

**Authors:** Raymond Cesaire, Agnès Lézin, Jean-Marie Péloponèse

**Affiliations:** 1 Laboratoire de Virologie–Immunologie, EA 4537, Centre Hospitalier Universitaire de Fort-de-France, Fort-de-France, Martinique, France; 2 Centre d’Études d’Agents Pathogènes et Biotechnologies pour la Santé, CNRS FRE 3689, Université Montpellier 1, Université Montpellier 2, Montpellier, France

## Introduction

The 17th International Conference on Human Retrovirology: HTLV and Related Retroviruses took place in Trois Ilets, Martinque (FWI) from June 18th to June 21th, 2015. The main topic covered during the meeting was human T-lymphotropic viruses (HTLVs) and was subdivided in distinct sessions: clinical research, animal models, immunology, molecular and cellular biology, and virology. The following abstracts were presented.

Five of the presented abstracts are not included in this supplement as they have been published elsewhere [1-5].
